# Physicians’ Attitudes Toward Transgender Individuals and Commitment to Treat Them by Religiosity

**DOI:** 10.3390/healthcare14121627

**Published:** 2026-06-09

**Authors:** Yulia Treister-Goltzman, Lior Zadok-Fridman

**Affiliations:** 1 Department of Family Medicine and Siaal Research Center for Family Practice and Primary Care, Haim Doron Division of Community Health, Faculty of Health Sciences, Ben-Gurion University of the Negev, Beer-Sheva 84105, Israel; 2 Clalit Health Services, Southern District, Beer-Sheva 84105, Israel; liorz.md@gmail.com

**Keywords:** transgender health, family physicians, attitudes, Transgender Attitudes and Beliefs Scale, religiosity

## Abstract

**Background**: This study aimed to examine the association between the three subscales of the Transgender Attitudes and Beliefs Scale and professional commitment to treat transgender patients, and to test whether these associations were related to the physician’s level of religiosity. **Methods**: A cross-sectional study of 109 family physicians was conducted using a self-administered questionnaire between October 2021 and July 2023 in the southern district of Clalit Healthcare Services. Confirmatory factor analysis and multi-group structural equation modeling were carried out. Main measures included TABS subscales, level of religiosity, and physicians’ commitment to treat transgender patients. **Results**: Only the ‘Human value’ subscale was a statistically significant positive predictor of professional commitment to treat transgender patients among the whole population of physicians and among the subgroup of physicians with high religiosity at β = 0.36 (95% Confidence interval (CI) 0.11 to 0.61, *p* = 0.005), and β = 0.57 (95% CI 0.20 to 0.95, *p* = 0.003), respectively. Among physicians with a low level of religiosity there was a significant positive effect of the ‘Interpersonal comfort’ subscale on the professional commitment to treat transgender patients at β = 3.31 (95% CI 1.66 to 4.95, *p* < 0.001). Although the ‘Sex/gender beliefs’ subscale showed a significant negative association in the primary model (β = −2.91, 95% CI −4.64 to −1.17, *p* = 0.001), sensitivity analyses suggested that this finding should be interpreted with caution because of the strong correlation between TABS domains. **Conclusions**: Different TABS domains were associated with physicians’ professional commitment to treat transgender patients across levels of religiosity. These findings can help tailor interventions more effectively to specific religiosity subgroups.

## 1. Introduction

Transgender individuals are a socially and medically vulnerable group [1]. Studies have shown that they have less education, lower income, and are more likely to be single with fewer children [2]. Their medical vulnerability stems from the need for special medical services such as gender-affirming care and hormone therapy, and special health insurance coverage [3]. In addition, transgender patients experience discrimination, personal bias, and negative attitudes from health care professionals [3] leading to a reticence on their part to seek medical, and even emergency services because they anticipate and fear negative attitudes and negative quality of care. The sum of these factors leads to health disparities and high morbidity and mortality rates among transgender individuals, compared to the general population [4,5].

The Transgender Attitude and Beliefs Scale (TABS) is a psychometrically sound, multi-dimensional instrument which is frequently used to assess attitudes towards transgender individuals in the general population as well as among medical [6,7,8]. TABS relates to three domains: (1) ‘Interpersonal comfort’, which measures the respondent’s level of comfort in daily interactions with transgender individuals, (2) ‘Sex/gender beliefs’, which assesses underlying beliefs regarding gender, and (3) ‘Human value’, which assesses an individual’s inherent value. This multidimensionality is intended to encompass multiple facets of attitudes and beliefs towards transgender individuals.

When assessing attitudes of the medical staff towards transgender patients, it is presumed that the score of the questionnaire, and of its subscales, demonstrates an implicit bias that could affect the treatment provided to transgender individuals. Implicit bias, also known as implicit prejudice or implicit attitude, is defined by the American Psychological Association as a negative attitude, of which one is not consciously aware, against a specific social group [9]. The evidence indicates that healthcare professionals exhibit the same level of implicit bias as the wider population [10]. Studies have found a positive relationship between the level of implicit bias and stereotypes and lower quality of care [10]. Most health professionals strive to be compassionate and egalitarian and display a high level of professional behavior [11].

The Israeli healthcare system operates within a complex sociocultural context that may shape physicians’ attitudes toward transgender patients. The medical workforce in Israel is ethnically diverse and includes a substantial proportion of Arab physicians, who are fully integrated into healthcare delivery across community and hospital settings, reflecting the broader multicultural composition of Israeli society [12,13]. At the same time, healthcare providers and patients operate within a society characterized by the coexistence of secular, Jewish religious, and Muslim communities, each of which may hold differing normative frameworks regarding gender identity and sexuality. Within Jewish religious discourse, perspectives on gender transition range from more permissive interpretations in specific medical contexts to traditional halakhic views grounded in binary gender concepts, while in Muslim communities, similar heterogeneity exists, with religious norms interacting with family, community, and social expectations regarding gender roles [14]. Within this broader context, LGBTQ+ individuals in Israel, including transgender people, have experienced increasing legal recognition and institutional visibility, alongside partial access to gender-affirming healthcare services. However, these advances coexist with persistent social stigma and variability in acceptance, particularly in peripheral regions [15,16,17]. These intersecting societal, cultural, and professional dynamics may influence physicians’ exposure to transgender patients, their perceived preparedness to provide care, and their attitudes toward professional responsibility in treating this population. This interpretation is consistent with minority stress and structural stigma frameworks, which emphasize the role of broader social environments in shaping health outcomes and healthcare interactions among sexual and gender minority populations [18,19].

Previous research has shown that levels of religiosity have been associated in some studies with attitudes toward transgender individuals, defined as the extent to which individuals are involved in their religion or integrate it into daily life [19,20]. Most physicians report that their religious beliefs influence their professional decisions. This influence is especially notable in challenging or difficult clinical situations [21,22]. Although attitudes toward transgender individuals among family physicians in Southern Israel have previously been described, including comparisons between residents and senior physicians [23], the associations between specific TABS domains and physicians’ professional commitment to treat transgender patients, and the potential moderating role of religiosity, have not been examined.

The aims of the present study were to examine the association between the three subscales of the TABS with physicians’ professional commitment to treat transgender patient as any other patient, and to test whether these associations were moderated by the level of religiosity. The first hypothesis of the present study was that the ‘Human value’ subscale of TABS would have stronger, than other subscales of TABS, association with professional commitment to treat a transgender patient with the same attention as for any other patient. The second hypothesis of the present study was that among physicians with a high religiosity level the positive association between the ‘Human value’ subscale of TABS and the professional commitment to treat transgender patients would be stronger than among physicians with low level of religiosity.

## 2. Methods

### 2.1. Procedures

This was a cross-sectional study. It was a part of a larger study that assessed attitudes of residents and senior physicians in Southern Israel towards transgender people and was conducted between October 2021 and July 2023. Findings from a previous analysis of the same survey dataset, comparing attitudes between residents and senior physicians have been published previously [23]. The present analysis addresses a different research question by examining the associations between the three domains of TABS and physicians’ professional commitment to treat transgender patients, as well as the moderating role of religiosity. The study population was comprised of family physicians who work in the southern district of Clalit Healthcare Services.

The study was conducted in accordance with the ethical standards set out in the 1964 Declaration of Helsinki, as revised in 2013. The Ethics Committee of Clalit Health Services, Israel, exempted the study from the requirement to obtain ethics approval and informed consent on 25 August 2020, as the study was an anonymous educational study among physicians. All participants agreed to participate in the study.

The study questionnaire was distributed among family physicians at staff meetings in the clinic, at continuous education programs in the Department of Family Medicine, conferences, and directly to physicians at their clinics. Two respondents were excluded from the present study due to more than 30% missing responses. For the remaining participants, missing data did not exceed one response.

### 2.2. Measures

The self-administered questionnaire included:

Demographic variables, including items on ethnicity and level of religiosity (self-reported).

Professional variables: status, previous acquaintance (professional and personal) with transgender individuals, and professional training.

**Figure A1:**
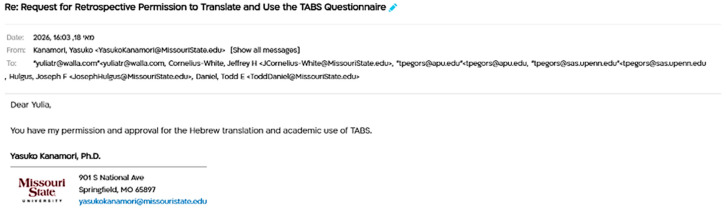
Author Permission for Hebrew Translation and Use of the ‘Transgender Attitudes and Beliefs Scale’.

The TABS questionnaire. The TABS is a free-to-use instrument for non-commercial academic research and has been translated into multiple languages [6,7,8]. Permission to use and translate the TABS questionnaire into Hebrew for the present study was obtained retrospectively from the original authors and is provided in Appendix A Figure A1. The subscales of the TABS questionnaire served as the main independent variables for this study. The ‘Interpersonal comfort’ domain of this questionnaire includes 14 items, for example ‘If I were introduced to a transgender person at a party, I would feel comfortable having a polite conversation with that person,’ the ‘Sex/gender beliefs’ domain consists of 10 items, for instance ‘A person who is not sure about being a man or woman is mentally ill,’ and the ‘Human value’ domain consists of 5 items, for example ‘Transgender individuals are valuable human beings regardless of how I feel about transgenderism.’

The questionnaire was translated to Hebrew using the backward translation method (Appendix A Table A1 and Table A2). Responses on TABS were rated on a 7-point Likert scale for each item and ranged between 1 ‘strongly disagree’ and 7 ‘strongly agree’. To minimize bias, the questionnaire includes a mix of positively and negatively formulated items (with reversed scoring). Higher scores indicate positive perceptions. The possible raw ranges of each of the domains of the questionnaire are: 14–98 for IC, 10–70 for ‘Sex/gender beliefs’, and 5–35 for ‘Human value’ [6,7].

The grouping variable of the level of religiosity was examined for its moderating effect on the association between the independent and dependent variables. The physicians answered the question: ‘How much does religion influence you and guide you in your everyday life?’. Those who answered ‘not at all’ or ‘partly’ were classified as low level of religiosity and those who answered ‘to a significant extent’ or ‘to a large extent’ with a high level of religiosity.

The dependent variable was a dichotomous answer to the question if you would treat transgender patient with the same attention and willingness to help as any other patient (‘yes’ or ‘no’).

### 2.3. Statistical Analysis

Statistical analyses were conducted using R software (version 4.3.3), ‘lavaan’ package [24,25]. Descriptive statistics were carried out and presented for the whole sample and by the grouping variable. Categorical variables such as sex, family status, and ethnicity are presented as frequencies and percentages. Continuous variables such as age are described as means and standard deviations (SD). Categorical variables were tested for differences by the chi-square test or Fisher’s exact test in accordance with the size of the cells. Differences in continuous variables were identified by *t*-test/Mann–Whitney rank-sum test.

In the first stage, a confirmatory factor analysis (CFA) was conducted to assess the stability of the factor structure of the Hebrew translation of the questionnaire. Convergent validity was assessed by measurement of model fit to the data, examining the standardized factor loadings, and average variance extracted (AVE) of each latent variable [26]. Construct reliability was assessed by ω (the proportion of variance shared between the items and the latent construct) [26]. Discriminant validity was assessed by constraining the correlations between each pair of the latent variables to one and comparing the model fit difference for significance [26].

The fit of the model was evaluated by the values of the comparative fit index (CFI), the Tucker Lewis index (TLI), and the root mean square error of approximation (RMSEA). CFI and TLI scores > 0.90 were interpreted as a good fit and >0.95 as a very good fit. An RMSEA score of <0.08 indicates a good fit and <0.05 a very good fit.

In the second stage, associations of the subscales of TABS with professional commitment to treat transgender individuals were tested in a structural equation model (SEM) using the weighted least squares mean and variance adjusted (WLSMV) estimator, which is appropriate for dichotomous outcome variables. First, a model without grouping was built. Then multi-group SEM across the levels of religiosity groups was created. Measurement invariance, an essential prerequisite for the multi-group SEM [27], was tested to verify equivalent construct measurements across different groups. The models’ fit and association coefficients were assessed, and differences in TABS subscale coefficients between low- and high-religiosity groups were tested for significance.

Missing data were minimal and limited to a small number of questionnaire items and were handled using pairwise deletion within the estimation procedure.

## 3. Results

Table 1 presents the baseline characteristics of the participants.

One hundred and nine physicians comprised the study sample. Their mean (SD) age was higher among those with a low level of religiosity than those with a high level at 45.41 (11.10) vs. 38.16 (12.36) (*p* = 0.003). Most of the respondents with a low level of religiosity were Jews (76.9%), while most Muslims (52.3%) had a high level (*p* < 0.001). Around 15% of the respondents had relatives/close acquaintances who were transgender individuals, close to 50% had treated transgender patients in the past, and close to 22% received some training to treat transgender patients, with no significant difference between the study groups for any item.

### 3.1. Confirmatory Factor Analysis of the Hebrew Translation of Transgender Attitude and Beliefs Scale

A first model, where the only permitted correlations were among the three factors, ‘Interpersonal comfort’, ‘Sex/gender beliefs’, and ‘Human value’, yielded a suboptimal model fit range: RMSEA = 0.10, TLI = 0.81, and CFI = 0.82. Modification indices (MI) were examined to consider possible error covariances. Several residual covariances suggested by MI were added to the model when conceptually justified by overlap in item wording, content, or social context. Most added residual covariances occurred between items within the same latent construct and reflected highly similar interpersonal or ideological situations. For example, correlations were added between Q1.1 and Q1.2, both reflecting general interpersonal comfort with transgender individuals, between Q1.3R and Q1.4R, both addressing discomfort in workplace interactions with transgender persons, and between Q2.2 and Q2.7, both concerning the legitimacy of gender identities beyond the male/female binary. In addition, a limited number of cross-factor residual covariances were specified between items belonging to the ‘Interpersonal comfort’ and ‘Sex/gender beliefs’ factors, which were themselves strongly correlated in the study sample. These cross-factor associations reflected overlapping constructs related to stigma-related attitudes, gender essentialism, and interpersonal social distance. Examples included covariance between Q1.6 (“I would feel comfortable if my next-door neighbor was transgender”) and Q2.1R (“A person who is not sure about being male or female is mentally ill”), as well as between Q1.10R (“If a transgender person asked to be my housemate, I would want to decline”) and Q2.8R (“All adults should identify as either male or female”). Another cross-factor covariance was specified between Q2.4R (“Whether a person is male or female depends strictly on their external sex-parts”) and Q3.1 (“Transgender individuals are valuable human beings regardless of how I feel about transgenderism”), reflecting overlap between biological essentialist beliefs and attitudes regarding the fundamental value of transgender individuals.

**Figure 1:**
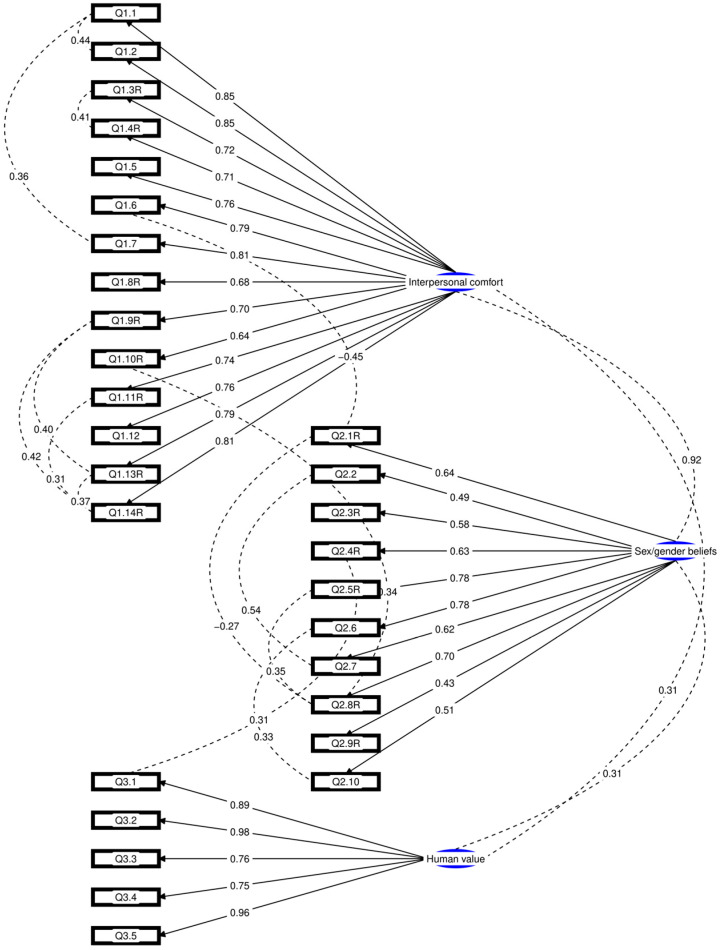
Confirmatory factor analysis of the Hebrew translation of transgender attitude and beliefs scale.

These residual covariances were introduced sequentially and retained only when theoretically meaningful and consistent with the conceptual structure of the TABS questionnaire, rather than solely on statistical grounds. All added covariances are reported in detail in Appendix A Table A3. A CFA with the revised parameter specifications yielded a better model fit: RMSEA = 0.08, TLI = 0.90, and CFI = 0.91 (Figure 1). Each of the 29 items had moderate to high factor loadings, ranging from 0.43 to 0.98, suggesting that the indicators were highly related to the purported factors. The AVE of the subscales was 0.59, 0.41 and 0.74 for the ‘Interpersonal comfort’, ‘Sex/gender beliefs’, and ‘Human value’ subscales, respectively.

The reliability estimate for each of the factors was high: ω = 0.95 for ‘Interpersonal comfort’, ω = 0.87 for ‘Sex/gender beliefs’, and ω = 0.93 for ‘Human value’, showing high internal consistency for each subscale. The values of factor correlations among the three subscales showed higher correlation between the ‘Interpersonal comfort’ and ‘Sex/gender beliefs’ subscales (r = 0.92), than between ‘Interpersonal comfort’ and ‘Human value’ (r = 0.31), and ‘Sex/gender beliefs’ and ‘Human value’ (r = 0.31) subscales. Discriminant validity testing showed that though the correlation between the ‘Interpersonal comfort’ and ‘Sex/gender beliefs’ subscales was high, they related to different constructs, since constraining the co-variance between them to one led to a significantly worse model fit (Δχ^2^ = 6.43, *p* = 0.011). Notably, the model fit indices and factor loadings obtained from the present Hebrew translation of the questionnaire were like those reported in the original CFA questionnaire validation by Kanamori et al. [6].

### 3.2. Association of the Transgender Attitude and Beliefs Scale Subscales with Professional Commitment to Treat Transgender Patients

Overall, 102 physicians (93.6%) reported that they would treat a transgender patient with the same attention and willingness to help as any other patient, whereas seven physicians (6.4%) reported that they would not.

**Figure 2:**
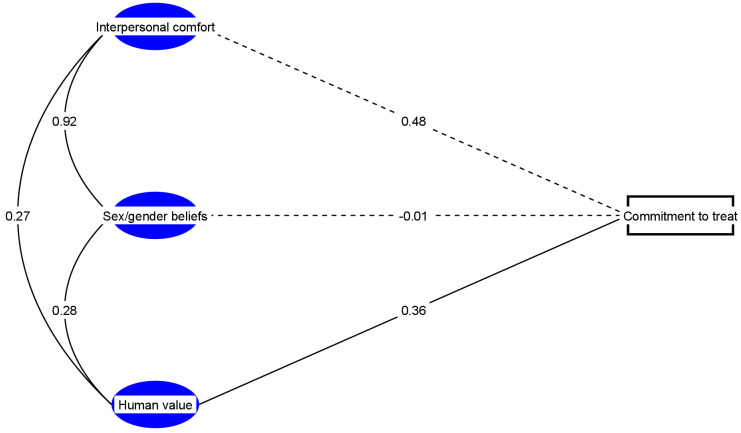
Association of subscales of the transgender attitude and beliefs scale questionnaire with physicians’ professional commitment to treat transgender patients. Solid lines indicate statistically significant paths; dashed lines indicate non-significant paths.

The SEM examined direct associations between the three TABS subscales (‘Interpersonal comfort’, ‘Sex/gender beliefs’, and ‘Human value’) and professional commitment to treat transgender patients. Each path coefficient represents the standardized effect of a given TABS domain on commitment to treat. The SEM for the whole sample adequately fit the data (χ^2^ = 180.68, df = 386, *p* = 1.00, RMSEA = 0.00, TLI = 1.09, CFI = 1.00) (Figure 2). Only the ‘Human value’ subscale was a statistically significant positive predictor of professional commitment to treat transgender patients β = 0.36 (95% CI 0.11 to 0.61, *p* = 0.005). The estimates of the associations of ‘Interpersonal comfort’ and ‘Sex/gender beliefs’ with professional commitment to treat were insignificant: β = 0.48 (95% CI −0.64 to 1.60, *p* = 0.397) and β = −0.01 (95% CI −1.13 to 1.12, *p* = 0.990), respectively.

### 3.3. Multi-Group Analysis by the Level of Religiosity

In the multigroup analysis, the three TABS subscales and professional commitment were estimated separately for physicians with low and high religiosity to assess potential moderation effects. Differences in path coefficients between groups indicate variation in the strength and direction of associations across religiosity levels. The measurement invariance model, which constrained the factor loadings to be equal between the two groups, was homogeneous with the unconstrained SEM (Δχ^2^ = 22.36, df = 26, *p* = 0.669). Since the measurement invariance requirement was satisfied, a multi-group analysis was used to assess the moderation effect of religiosity level. Fit indices of the multi-group SEM were also excellent (χ^2^ = 341.10, df = 772, *p* = 1.00, RMSEA = 0.00, TLI = 1.18, CFI = 1.00).

Table 2 shows the path coefficients between variables for each group and comparison of the path coefficients across the groups, obtained by the χ^2^ difference test.

**Figure 3:**
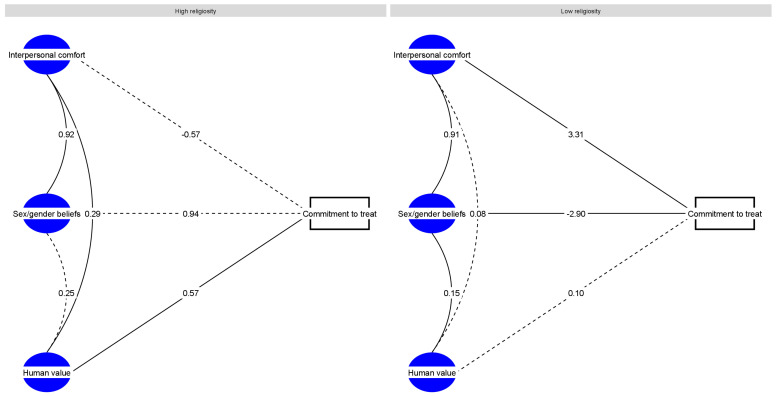
Multi-group analysis of the association of subscales of the transgender attitude and beliefs scale with physicians’ professional commitment to treat transgender patients. Solid lines indicate statistically significant paths; dashed lines indicate non-significant paths.

Among physicians with a low level of religiosity a significant positive effect of ‘Interpersonal comfort’ and a significant negative effect of ‘Sex/gender beliefs’ on the professional commitment to treat transgender patients were demonstrated β = 3.31 (95% CI 1.66 to 4.95, *p* < 0.001), and −2.91 (−4.64 to −1.17, *p* = 0.001), respectively (Figure 3). The association of ‘Human value’ with this professional commitment was nonsignificant. Among physicians with high religiosity the only significant effect on the dependent variable was for ‘Human value’ (β = 0.57 (95% CI 0.20 to 0.95, *p* = 0.003). The differences between the groups were significant for the paths of ‘Interpersonal comfort’ (*p* = 0.002) and ‘Sex/gender beliefs’ (*p* = 0.005).

### 3.4. Sensitivity Analyses

Given baseline differences between religiosity groups in age, ethnicity, professional status, and seniority, sensitivity analyses were conducted to assess the robustness of the primary findings after adjustment for socio-demographic characteristics. Because age, seniority as a physician, and professional status were strongly associated and reflected overlapping aspects of professional experience, only seniority as a physician was retained in the adjusted analyses in order to reduce redundancy among covariates. Due to the small subgroup sample sizes and the complexity of the multigroup SEM with a categorical outcome variable, stable estimation of fully adjusted multigroup models was not feasible. Therefore, adjusted SEM analyses were performed in the overall sample. In the first adjusted model, ethnicity and seniority as a physician were included as observed covariates predicting the outcome variable, while the measurement and structural components of the model remained unchanged. The adjusted model demonstrated good fit to the data (RMSEA = 0.03, TLI = 0.97, CFI = 0.97), and the pattern of findings remained similar to the unadjusted whole-sample SEM, with ‘Human value’ remaining the only significant variable associated with professional commitment to treat transgender patients. In an additional adjusted model, religiosity was also included as an observed covariate. This model also demonstrated good fit to the data (RMSEA = 0.05, TLI = 0.96, CFI = 0.96). In this analysis, ‘Human value’ remained significantly associated with the outcome variable, while ‘Interpersonal comfort’ emerged as a borderline significant variable after adjustment for religiosity and socio-demographic variables (Appendix A Table A4). These findings are consistent with the multigroup analyses and suggest that religiosity may influence the associations between TABS domains and professional commitment to treat transgender patients.

Because the ‘Interpersonal comfort’ and ‘Sex/gender beliefs’ domains were strongly correlated, additional sensitivity analyses were conducted to evaluate the stability of the multigroup SEM findings. Two alternative models were examined in which each of these domains was alternately excluded from the structural component of the model while retained in the measurement model (Appendix A Table A5). Among physicians with high religiosity, the positive association between ‘Human value’ and professional commitment to treat transgender patients remained virtually unchanged across both models (β = 0.52 (*p* = 0.002) and β = 0.53 (*p* = 0.001), respectively). Among physicians with low religiosity, ‘Human value’ remained non-significant in both models, whereas either ‘Interpersonal comfort’ or ‘Sex/gender beliefs’ emerged as significant predictors when examined separately. These findings indicate that the strong correlation between ‘Interpersonal comfort’ and ‘Sex/gender beliefs’ primarily influences the estimation of their individual effects, while the association between ‘Human value’ and professional commitment to treat transgender patients among highly religious physicians remains stable. Overall, the consistency of the findings across alternative model specifications supports the robustness of the main results.

## 4. Discussion

The first hypothesis of this study, that the ‘Human value’ subscale of the TABS questionnaire would have a stronger association than other subscales with professional commitment to treat transgender patient as any other patient was partially confirmed. In the overall SEM, ‘Human value’ was the only TABS domain significantly associated with the dependent variable. In the multigroup analyses, however, the pattern differed according to religiosity level, with ‘Human value’ remaining significantly associated with professional commitment only among physicians with high religiosity. The professional behavior of physicians is a way of acting, by which individual physicians realize their medical professionalism [28]. As formulated by J. Cohen: “Humanism provides the passion that animates authentic professionalism” [28]. In the context of medical practice, humanistic physicians are intuitively strongly motivated to adhere to the traditional virtues and expectations of their calling. Our study provides further proof that humanism and human value are the main factors associated with professional commitment in the special case of transgender patients.

The second hypothesis, that the level of religiosity moderates the association between the ‘Interpersonal comfort’, ‘Sex/gender beliefs’ and ‘Human value’ subscales and professional commitment to treat transgender patients, was also confirmed. In a nation-wide study of American physicians’ religious values, 55% of physicians reported that their religious beliefs influenced their practice of medicine [29]. Among physicians with high religiosity, as in the general population of physicians, the ‘Human value’ subscale was significantly associated with professional commitment to treat transgender patient as any other person. Often, religious values are not very distinct from common morality and professional morality [30]. Those are empathy and compassion, sense of duty, precision, meticulousness, continuous improvement, and putting patients’ interests above those of their physicians [31].

Opinions and decision making in clinical practice can be conceptualized using a dual process theory, which involves type 1 and type 2 processes [32]. Type 1 processes are fast and intuitive. They are often known as mental shortcuts and are responsible for rapid decision making. In contrast, type 2 processes are slower, analytic, and require more cognitive resources. It is thought that our biases are formed in early life from reinforcement of stereotypes. And while religious beliefs are probably greatly responsible for the type 1 process of decision making, education and experience have an important role in the growth and development of professional values [33] and hence could enhance type 2 decision processes. This framework may provide a useful way to interpret how professional judgments and attitudes could be influenced by both automatic and reflective processes. However, the present study did not directly test this theoretical model, and its application here should be considered speculative.

Interestingly, among physicians with a low level of religiosity, ‘Sex/gender beliefs’ and ‘Interpersonal comfort’ were the factors associated with professional commitment to treat transgender patients as any other patient. In the primary multigroup SEM, ‘Sex/gender beliefs’ showed a negative association with professional commitment. However, this finding should be interpreted cautiously. The original structure of the TABS questionnaire was retained in order to preserve the validated measurement model and facilitate comparison with previous studies. Nevertheless, in our sample, the correlation between the ‘Sex/gender beliefs’ and ‘Interpersonal comfort’ domains was very high (r = 0.92), indicating substantial shared variance between the two constructs. Sensitivity analyses demonstrated that when ‘Interpersonal comfort’ was excluded from the model, the association between ‘Sex/gender beliefs’ and professional commitment became positive and remained statistically significant. These findings suggest that the negative coefficient observed in the primary model may reflect a statistical suppression effect resulting from the high correlation between the two domains rather than a true inverse association. Therefore, among physicians with low religiosity, more positive ‘Sex/gender beliefs’ and greater ‘Interpersonal comfort’ both appear to be associated with greater professional commitment to treat transgender patients. And while interventions aimed to change beliefs on transgender health issues didn’t succeed in improving beliefs, the attitude, comfort level, and knowledge did improve [34].

The strong association between ‘Interpersonal comfort’ and professional commitment is consistent with previous studies showing that exposure to transgender patients, clinical experience, and iterative feedback can improve physicians’ comfort and confidence in providing care for transgender individuals [35,36]. A recent systematic review showed that different short term and long-term interventions, at theoretical, educational and practical levels, have provided a significant positive increase in attitudes, ‘Interpersonal comfort’, confidence, and knowledge with respect to transgender health issues and care for transgender patients [37]. Taken together, these findings suggest that both attitudinal and experiential factors may contribute to physicians’ professional commitment to provide equitable care for transgender patients.

In the context of the complex sociocultural, religious, and institutional environment surrounding transgender healthcare in Israel, the present study contributes to the understanding of one important aspect of this broader issue by focusing specifically on physicians’ professional commitment to treat transgender patients and the attitudinal domains associated with it. The findings suggest that different components of attitudes toward transgender individuals may be associated with professional commitment in different ways according to the physician’s level of religiosity, highlighting the importance of considering sociocultural context when addressing transgender healthcare disparities. It should also be noted that self-reported professional commitment to treat transgender patients may reflect factors beyond attitudes alone, including perceived preparedness, clinical experience, and knowledge regarding transgender healthcare. Consequently, the outcome measure should not be interpreted solely as an indicator of bias or prejudice. Future studies should examine the relative contributions of attitudes, training, knowledge, and clinical competence to physicians’ willingness and preparedness to provide care for transgender patients. In a healthcare system characterized by multiculturalism, religious diversity, and ongoing social changes regarding gender and sexuality, identifying factors associated with professional commitment to care may help inform educational and professional interventions aimed at improving healthcare experiences for transgender patients.

### Limitations and Strengths

The first limitation of our study is its questionnaire-based design with possible recall /habituation biases. Although the survey was completed anonymously, which may have reduced the tendency toward socially desirable responding, such bias cannot be entirely eliminated in studies addressing sensitive attitudinal topics. The cross-sectional design assessed associations at a single point in time only, rather than causal or temporal relationships between variables. However, questionnaires are still the most acceptable and feasible way to assess the attitudes of physicians. The regional nature of the study, with a specific demographic profile of physicians in the peripheral southern region of Israel, limits the generalizability of the findings. Several additional methodological limitations should be acknowledged. First, religiosity was assessed using a single self-report item, which may not fully capture its multidimensional nature. Second, the dependent variable was dichotomous, which reduces a complex construct (professional commitment to treat transgender patients) into a simplified yes/no measure and may limit the ability to capture gradations in physicians’ attitudes and intentions. In addition, the outcome variable showed limited variability, with only 6.4% of physicians reporting that they would not treat a transgender patient with the same attention and willingness as any other patient. This imbalance may have reduced statistical power and the precision of parameter estimates. Some items of the TABS questionnaire may be interpreted differently by respondents. For example, responses to items concerning gender identity and mental health may reflect varying interpretations of the item wording rather than solely attitudes toward transgender individuals. Although the present analyses were conducted at the latent construct level, such variation in item interpretation cannot be completely excluded. The relatively small sample size (N = 109), particularly within multigroup SEM, may limit model stability, reduce statistical power, and increase uncertainty in parameter estimation. Fourth, the very high correlation between the ‘Interpersonal comfort’ and ‘Sex/gender beliefs’ subscales (r = 0.92) may have affected the stability of individual regression coefficients. Although sensitivity analyses were conducted to explore the possibility of a suppression effect, results involving these domains should be interpreted with caution. We did not examine potential nonlinear or higher-order interaction effects due to sample size constraints and model complexity; these may be explored in future research. Although sensitivity analyses were conducted, residual confounding by ethnicity and other socio-demographic variables within religiosity groups cannot be fully excluded. Finally, the CFA also included theoretically justified modifications based on modification indices. Although these refinements were conceptually grounded and consistent with overlap in item content, they may limit strict cross-study comparability and should, therefore, be interpreted with caution.

## 5. Conclusions

‘Human value’ is the main factor associated with professional commitment to treat transgender patients among physicians with a high religiosity level. ‘Sex/gender beliefs’ and ‘Interpersonal comfort’ were influential factors among physicians with a low religiosity level, although the associations involving these highly correlated domains should be interpreted cautiously. As we discussed above, ‘Human value’ is not based solely on implicit stereotypes, as it can be improved by interventions. The results of the present study can help focus interventions on subgroups of physicians with different levels of religiosity in a more efficient way.

## Figures and Tables

**Table 1:** Baseline characteristics of study participants.

Variable	Total (N = 109) Mean ± SD or N (%)	Level of Religiosity		
		Low (N = 65) Mean ± SD or N (%)	High (N = 44) Mean ± SD or N (%)	*p*-Value
**Age**	42.65 ± 12.20	45.41 ± 11.10	38.16 ± 12.36	0.003
**Sex**				
Male	55 (50.4)	30 (46.2)	25 (56.8)	0.270
Female	54 (49.5)	35 (53.8)	19 (43.2)	
**Gender**				
Male	55 (50.4)	30 (46.2)	25 (56.8)	0.270
Female	54 (49.5)	35 (53.8)	19 (43.2)	
Transgender	0 (0.00)	0 (0.0)	0 (0.0)	
**Ethnicity**				
Jew	70 (64.2)	50 (76.9)	20 (45.4)	<0.001
Muslim Arab	29 (26.6)	6 (9.2)	23 (52.3)	
Other	10 (9.2)	9 (13.8)	1 (2.3)	
**Family status**				
Lives with a spouse	88 (80.7)	55 (84.6)	33 (75.0)	0.306
Doesn’t live with a spouse	21 (19.3)	10 (15.4)	11 (25.0)	
**Country of birth**				
Israel	69 (63.3)	34 (52.3)	35 (79.5)	0.006
Abroad	40 (36.7)	31 (47.7)	9 (20.5)	
**Professional status**				
Senior	55 (50.5)	42 (64.6)	13 (29.5)	0.002
Resident	54 (49.5)	23 (35.4)	31 (70.5)	
**Seniority as a physician**, years	13.94 ± 12.20	16.74 ± 12.38	9.43 ± 12.13	0.003
**Have relatives/close acquaintances who are transgender people?**				
Yes	17 (15.6)	11 (16.9)	6 (13.6)	0.819
No	92 (84.4)	54 (83.1)	38 (86.4)	
**Ever treated transgender people?**				
Yes	54 (49.5)	35 (53.8)	19 (43.2)	0.321
No	55 (50.5)	30 (46.2)	25 (56.8)	
**Received training to treat transgender people?**				
Yes	24 (22.0)	13 (20.0)	11 (25.0)	0.734
No	85 (78.0)	52 (80.0)	33 (75.0)	

SD—Standard deviation.

**Table 2:** Multi-group analysis of the association of transgender attitude and beliefs scale subscales with professional commitment to treat transgender patients.

Variable Path	β (95% CI)	*p*-Value	β (95% CI)	*p*-Value	|Δ β|	*p*-Value
	Low Level of Religiosity		High Level of Religiosity		Path Difference	
IC→CT	3.31 (1.66 to 4.95)	<0.001	−0.57 (−2.46 to 1.33)	0.558	3.88	0.002
SGB→CT	−2.91 (−4.64 to −1.17)	0.001	0.94 (−1.07 to 2.94)	0.361	3.85	0.005
HV→CT	0.10 (−0.35 to 0.55)	0.663	0.57 (0.20 to 0.95)	0.003	0.49	0.113

IC—‘Interpersonal comfort’, SGB—‘Sex/gender beliefs’, HV—‘Human value’, CT—Professional commitment to treat transgender patient as any other patient, CI—Confidence intervals.

## Data Availability

Data available on request due to ethical and legal.

## Abbreviations

The following abbreviations are used in this manuscript:

TABS	The Transgender Attitude and Beliefs Scale
SD	Standard Deviations
CFA	Confirmatory Factor Analysis
AVE	Average Variance Extracted
CFI	Comparative Fit Index
TLI	The Tucker Lewis Index
RMSEA	The Root Mean Square Error of Approximation
SEM	Structural Equation Model
MI	Modification Indices

## Appendix A

**Table A1:** The original ‘Transgender Attitudes and Beliefs Scale’.

Please Answer Each Item by Marking One Box	1 Strongly Disagree	2	3	4	5	6	7 Strongly Agree
Interpersonal comfort subscale							
1. I would feel comfortable having a transgender person into my home for a meal.							
2. I would be comfortable being in a group of transgender individuals.							
R. 3. I would be uncomfortable if my boss was transgender.							
R. 4. I would feel uncomfortable working closely with a transgender person in my workplace.							
5. If I knew someone was transgender, I would still be open to forming a friendship with that person.							
6. I would feel comfortable if my next-door neighbor was transgender.							
7. If my child brought home a transgender friend, I would be comfortable having that person in my home.							
R. 8. I would be upset if someone I’d known for a long time revealed that they used to be another gender.							
R. 9. If I knew someone was transgender, I would tend to avoid that person.							
R. 10. If a transgender person asked to be my housemate, I would want to decline.							
R. 11. I would feel uncomfortable finding out that I was alone with a transgender person.							
12. I would be comfortable working for a company that welcomes transgender individuals.							
R. 13. If someone I knew revealed to me that they were transgender, I would probably no longer be as close to that person.							
R. 14. If I found out my doctor was transgender, I would want to seek another doctor.							
Sex/Gender beliefs subscale							
R. 1. A person who is not sure about being male or female is mentally ill.							
2. Whether a person is male or female depends upon whether they feel male or female.							
R. 3. If you are born male, nothing you do will change that.							
R. 4. Whether a person is male or female depends strictly on their external sex-parts.							
R 5. Humanity is only male or female; there is nothing in between.							
6. If a transgender person identifies as female, she should have the right to marry a man.							
7. Although most of humanity is male or female, there are also identities in between.							
8. R. All adults should identify as either male or female.							
9. R. A child born with ambiguous sex-parts should be assigned to be either male or female.							
10. A person does not have to be clearly male or female to be normal and healthy.							
Human value subscale							
1. Transgender individuals are valuable human beings regardless of how I feel about transgenderism.							
2. Transgender individuals should be treated with the same respect and dignity as any other person.							
3. I would find it highly objectionable to see a transgender person being teased or mistreated.							
4. Transgender individuals are human beings with their own struggles, just like the rest of us.							
5. Transgender individuals should have the same access to housing as any other person							

**Table A2:** Hebrew version of Transgender Attitudes and Beliefs Scale.

7 מסכים מאוד	6	5	4	3	2	1 מאוד לא מסכים	אנא השב לכל פריט על ידי סימון תיבה אחת
							א. נוחות אישית
							א.1. ארגיש בנוח לארח בן אדם טרנסג’נדר בארוחה בביתי
							א.2. ארגיש בנוח עם בקבוצת אנשים טרנסג’נדרים
							א.3. לא היתי מרגיש בנוח עם המנהל שלי היה טרנסג’נדר
							א.4. לא היתי מרגיש בנוח לעבוד בסמיכות עם בן אדם-טרנסג’נדר במקום העבודה
							א.5. אם היתי יודע שבן אדם מסויים טרנסג’נדר, עדיין היתי פתוח ליצור יחסי חברות עם האיש הזה
							א.6. היתי מרגיש בנוח עם השכן ה”שכן בדלת לידי” היה טרנסג’נדר
							א.7. אם הבן שלי היה מביא הביתה חבר טרנסג’נדר היתי מרגיש בנוח לארח אותו
							א.8. היתי עצוב/כועס אם מישהו שאני מכיר הרבה זמן היה מגלה לי שהיה שייך בעבר למין אחר
							א.9. אם היתי יודע שמישהו טרנסג’נדר היתי נמנע מקרבתו
							א.10. אם איש טרנסג’נדר היה רוצה להיות שותף בדירה שלי היתי מסרב
							א.11. היתי מרגיש לא נוח למצוא את עצמי לבד עם איש טרנסג’נדר
							א.12. היתי מרגיש נוח לעבוד בחברה שמקבלת בברכה אנשים טרנסג’נדרים
							א.13. אם מישהו שאני מכיר היה מגלה לי שהוא טרנסג’נדר, זה כנראה יחליש את הקרבה בינינו
							א.14. אם היתי מגלה שהרופא שלי טרנסג’נדר, היתי רוצה לחפש רופא אחר
							ב. אמונות הקשורות למין/מגדר
							ב.1. בן אדם שלא בטוח האם הוא גבר או אישה הינו חולה נפש
							ב.2. היותו של אדם גבר או אישה תלוי באם הוא מרגיש גבר או אישה
							ב.3. אם נולדת גבר, כל מה שתעשה לא ישנה זאת
							ב.4. היותו של בן אדם גבר או אישה תלוי רק באברי המין החיצוניים שלו
							ב.5. אדם יכול להיות רק גבר או אישה, אין משהו אחר באמצע.
							ב.6. אם בן אדם טרנסג’נדר מזדהה כאישה יש לה את הזכות להנשא לגבר
							ב.7. למרות שרוב האנשים הינם או גברים או נשים ישנם אנשים שהם באמצע
							ב.8. כל המבוגרים צריכים להזדהות או כגבר או כאישה
							ב.9. אם ילד נולד עם איברי מין לא ברורים (נשיים/גבריים) צריכים להגדיר אותו כגבר או אישה בלבד
							ב.10. בן אדם לא חייב להיות אישה או גבר בלבד כדי להיות נורמאלי ובריא
							ג. הערך האנושי
							ג.1. אנשים טרנסג’נדרים הינם בני אנוש בעלי ערך ללא קשר איך אני מרגיש לגבי טרנסגנדרים
							ג.2. צריך להתיחס לטרנסג’נדרים עם אותו כבוד כמו לכל בן אדם אחר
							ג.3. היתי מתנגד להצקה או יחס רע כלפי אנשים טרנסג’נדרים
							ג.4. אנשים טרנסג’נדרים זה אנשים עם אתגרים אישיים, בדיוק כמו כולם
							ג.5. לאנשים טרנסגנדרים יש זכות לדיור כמו לכל בן אדם אחר.

**Table A3:** List of residual covariances added to the confirmatory factor analysis (CFA) model.

1.	Q1.1 ~~ Q1.2
2.	Q1.1 ~~ Q1.7
3.	Q1.3R ~~ Q1.4R
4.	Q1.9R ~~ Q1.13R
5.	Q1.11R ~~ Q1.14R
6.	Q1.6 ~~ Q2.1R
7.	Q2.4R ~~ Q3.1
8.	Q1.9R ~~ Q1.14R
9.	Q1.10R ~~ Q2.8R
10.	Q1.13R ~~ Q1.14R
11.	Q2.1R ~~ Q2.8R
12.	Q2.2 ~~ Q2.7
13.	Q2.5R ~~ Q2.8R
14.	Q2.6 ~~ Q2.10

**Table A4:** Sensitivity analyses evaluating the robustness of structural equation modeling results with covariate adjustment.

	Adjusted for Ethnicity and Seniority		Adjusted for Ethnicity, Seniority and Religiosity	
Variable Path	β (95% CI)	*p*-Value	β (95% CI)	*p*-Value
IC→CT	0.48 (−0.09 to 1.04)	0.097	0.47 (−0.01 to 0.95)	0.053
SGB→CT	−0.10 (−0.74 to 0.54)	0.766	−0.13 (−0.69 to 0.42)	0.636
HV→CT	0.36 (0.12 to 0.59)	0.003	0.31 (0.11 to 0.51)	0.002

IC—‘Interpersonal comfort’, SGB—‘Sex/gender beliefs’, HV—‘Human value’, CT—Professional commitment to treat transgender patient as any other patient, CI—Confidence intervals.

**Table A5:** Sensitivity analyses evaluating the robustness of multigroup structural equation modeling results after alternative specification of highly correlated attitudinal domains.

Variable Path	Low Religiosity β (95% CI)	*p*-Value	High Religiosity β (95% CI)	*p*-Value
**SGB excluded from the structural model**				
HV→CT	−0.27 (−0.63 to 0.09)	0.138	0.52 (0.21 to 0.83)	0.001
IC→CT	0.59 (0.42 to 0.76)	<0.001	0.34 (0.06 to 0.63)	0.019
**IC excluded from the structural model**				
HV→CT	−0.34 (−0.71 to 0.03)	0.071	0.53 (0.19 to 0.86)	0.002
SGB→CT	0.57 (0.40 to 0.75)	<0.001	0.36 (0.06 to 0.67)	0.019

IC—‘Interpersonal comfort’, SGB—‘Sex/gender beliefs’, HV—‘Human value’, CT—Professional commitment to treat transgender patient as any other patient, CI—Confidence intervals.
